# Multi-omics analysis reveals PUS1 triggered malignancy and correlated with immune infiltrates in NSCLC

**DOI:** 10.18632/aging.205169

**Published:** 2023-11-02

**Authors:** Yonghuang Tan, Zhaotong Wang, Yingzhao Wang, Xiaolu Tian, Yunru Huang, Guoyong Wu, Jianjun Lu

**Affiliations:** 1 Department of Thoracic Surgery, The First Affiliated Hospital, Sun Yat-sen University, Guangzhou, Guangdong 510080, China; 2 Guangdong Key Laboratory of Chiral Molecule and Drug Discovery, and Guangdong Provincial Key Laboratory of New Drug Design and Evaluation, School of Pharmaceutical Sciences, Sun Yat-sen University, Guangzhou, Guangdong 510006, China

**Keywords:** lung cancer, PUS1, immune microenvironment

## Abstract

Non-small cell lung cancer (NSCLC) is the main pathological type of lung cancer. In this study, multi-omics analysis revealed a significant increase of pseudouridine synthase 1 (PUS1) in NSCLC and the high expression of PUS1 was associated with shorter OS (Overall Survival), PFS (Progression Free Survival), and PPS (Post Progression Survival) of NSCLC patients. Clinical subgroup analysis showed that PUS1 may be involved in the occurrence and development of NSCLC. Besides, TIMER, ESTIMATE, and IPS analysis suggested that PUS1 expression was associated with immune cell infiltration, and the expression of PUS1 was significantly negatively correlated with DC cell infiltration. GESA analysis also indicated PUS1 may involve in DNA_REPAIR, E2F_TARGETS, MYC_TARGETS_V2, G2M_CHECKPOINT and MYC_TARGETS_V1 pathways and triggered NSCLC malignancy through MCM5 or XPO1. Furthermore, PUS1 may be a potential target for NSCLC therapy.

## INTRODUCTION

Lung cancer is the second most commonly diagnosed cancer, and, leading the cause of cancer deaths among cancer types, it causes estimated 1.79 million deaths per year. [1]. Non-small cell lung cancer (NSCLC) is the main pathological type of lung cancer, encompassing approximately 85% of cases. Among NSCLC, lung adenocarcinoma (LUAD) and lung squamous cell carcinoma (LUSC) are the main pathological types [2]. Despite the continuous progress in lung cancer treatment, the prognosis for patients with inoperable lung cancer remains poor, with a 5-year survival rate of only 13–60% [3, 4]. It is urgent to explore and understand the molecular mechanism of the development and progression of NSCLC to improve the prognosis of patients with NSCLC.

More than 100 different types of post-synthesis modification have been shown to be presented on RNA [5]. Pseudouridine (Ψ), an isomer of uridine, represents the most abundant and widespread type of RNA epigenetic modification across all domains of life [6, 7]. In eukaryotes, Ψ can be catalyzed via two distinct mechanisms: an RNA-guided mechanism that involves the ribonucleoproteins of the box H/ACA class (DCK1) [8], and the other is an RNA-independent mechanism that involves pseudouridine synthases (PUSs) [9].

In human race, there are 13 “writers” for pseudouridine, including PUS1, PUS3, PUS7, PUS10, PUS7L, PUSL1, TRUB1, TRUB2, RPUSD1-4 and DKC1. The box H/ACA small ribonucleoproteins (box H/ACA small ribonucleoproteins) consisting of DKC1, Nhp2, Nop10, Gar1 and snoRNAs. In this complex, H/ACA RNA exerts enzymatic activity by primarily binding to the substrate rRNA. In the RNA-independent pathway, pseudouridine synthases such as PUS1 and PUS7 can directly catalyze Ψ by recognizing the secondary structure or primary sequence of the substrate RNA. Previous studies have found that Ψ affected RNA processing, translation, and RNA degradation [10–14]. However, the biological mechanism of Ψ remains poorly understood.

In recent years, the role of Ψ in cancer-related RNA processes has begun to be revealed [15]. DKC1 is associated with several cancers such as colorectal cancer [16, 17], glioblastoma [18], prostate cancer [19], breast cancer [20, 21], lung adenocarcinoma [22]. Downregulation of DKC1 could induce telomere-related cell senescence and apoptosis in LUAD. Besides, PUS7 was also proved to be upregulated in colorectal cancer [23], and glioblastoma stem cells [24] and correlated with the malignancy. Previous studies have demonstrated that PUS1, PUS7 and RPUSD1 induced pre-mRNA Ψ modifications regulated splicing and pre-mRNA processing in human hepatocellular carcinoma cells [13]. Studies have shown that PUS1 is a prognostic factor in liver cancer and breast cancer [25, 26]. Ψ plays a key role in tumors, especially in NSCLC, however, the biological roles of Ψ and PUSs in NSCLC remain unclear.

In the present study, multi-omics analysis revealed a significant increase of pseudouridine synthase 1 (PUS1) in NSCLC and the high expression of PUS1 was associated with shorter OS (Overall Survival), PFS (Progression Free Survival), and PPS (Post Progression Survival) of NSCLC patients. Clinical subgroup analysis showed that PUS1 may be involved in the occurrence and development of NSCLC. Besides, IPS analysis suggested that PUS1 expression was associated with immune cell infiltration. In addition, GESA analysis indicated PUS1 may be involved in DNA_REPAIR, E2F_TARGETS, MYC_TARGETS_V2, G2M_CHECKPOINT, and MYC_TARGETS_V1 pathways and trigger NSCLC malignancy. In conclusion, Ψ has emerged as a potential player in cancer processes and PUS1 may be a potential therapy target and novel biomarker in NSCLC.

## MATERIALS AND METHODS

### The expression analysis of pseudouridine synthases

TGCA_LUAD and TCGA_LUSC databases were downloaded and mined from The Cancer Genome Atlas (TCGA) for the fold-change analysis with thresholds of |Fold-change|>2; *P*-value < 0.05 after the data was standardized by R Studio, 13 pseudouridine synthase fold-change analysis results were extracted. Two lung cancer proteome datasets (study: CPTAC LSCC Discovery Study and CPTAC LUAD Discovery Study) [27, 28] were downloaded from the National Cancer Institute’s Clinical Proteomic Tumor Analysis Consortium (CPTAC, https://pdc.cancer.gov/pdc).

### GEO datasets processing

The GSE30219, GSE19188, GSE31210, GSE31547, GSE40791, GSE81089, GSE5364, GSE11117, GSE43458, GSE44077, GSE60052, GSE103512, GSE101929, GSE87340, GSE40419, GSE75037, GSE41271, GSE68465, GSE5828, GSE43580 and GSE79210 matrix and platform information were downloaded from the GEO (http://www.ncbi.nlm.nih.gov/geo). All the expression matrix has been normalized.

### Survival analysis in Kaplan-Meier plotter

Kaplan-Meier plotter (http://kmplot.com/analysis/) is capable of assessing the correlation between the expressions of 30k genes [29]. Univariate Cox regression analysis of 13 PUSs, 218670_at (PUS1), 221277_s_at (PUS3), 229362_at (PUS10), 218984_at (PUS7), 229751_s_at (PUS7L), 228733_at (PUSL1), 226339_at (TRUB1), 223109_at (TRUB2), 201479_at (DKC), 226078_at (RPUSD1), 221940_at (RPUSD2), 225743_at (RPUSD3), 225398_at (RPUSD4), were analyzed in lung cancer module. The patients were split by the “Auto select the best cutoff” option. After excluding the outlier arrays, the OS (Overall survival), PPS (Post progression survival), and FPS (First progression survival) Kaplan-Meier survival curves were obtained. Besides, the Kaplan-Meier survival analysis of PUS1 based on GSE15701 (*N* = 235), GSE68465 (*N* = 442), and GSE30219 (*N* = 293) were drawn separately.

### Correlation analysis in UALCAN

UALCAN (https://ualcan.path.uab.edu/) was used to further confirm the significantly correlated genes with PUS1 in the “TCGA” module [30]. After entering Gene Symbol (PUS1), the Correlation analysis module was selected and the analysis results were downloaded in LUAD or LUSC respectively. PUS1-correlated genes with extremely low expression (Median TPM < 0.5) are filtered out of the list, and *P*-values < 0.05 were included in the set. 677 (PUS1_Cor) genes were found respectively in LUAD and LUSC.

### GSEA pathway enrichment analysis

TGCA_LUAD and LUSC databases were analyzed by GSEA. The patients were divided into PUS1 high and PUS1 low according to the PUS1 expression (Cutoff: median), in GSEA analysis using hallmark gene sets. *P* < 0.05 was considered to be statistically significant.

### Potential Ψ modification target processing

PUS1 Ψ targets in eukaryotic cells were confirmed by several papers and accessed from previous studies [13]. To find the potential PUS1-dependent Ψ targets in NSCLC, an intersection analysis of the PUS1-depended Ψ modification genes, A549_Ψ modification genes [31], and PUS1-correlated genes were applied respectively.

### Immune infiltration analysis in NSCLC

Several analysis websites on immune infiltration were used to analyze the extent of immune infiltration of PUS1 in NSCLC by using TCGA-LUAD and LUSC. The correlations of PUS1 expression with immune infiltration levels in LUAD and LUSC were analyzed by TIMER [32]. ESTIMATE was used to analyze the relationship between PUS1 expression and stromal and immune infiltration scores [33]. IPS score was used to assess the correlation between PUS1 and tumor immunogenicity in NSCLC [34].

### Cell culture and cell transfection

The A549 cell lines were purchased from the American Type Culture Collection (ATCC, Manassas, VA, USA). Cells were cultured and maintained in DMEM supplemented with 10% fetal bovine serum (FBS), 100 U/ml penicillin, and 100 μg/ml streptomycin (Cat. No: 164210-50, Procell, China). Cells were incubated in a humidified chamber at 37°C in 95% air and 5% CO_2_.

The PUS1–siRNA (siPUS1–01: GCCAGAGCTTCATGATGCA; siPUS1–02: GTCGGGTCCTCACAATTCA, RiboBio, China). The transfection was performed when cells had grown to 50–60% according to the protocol (lp8000, C0533-1.5 ml, Beyotime, China).

### RT-qPCR

RNA extraction and real-time PCR for gene expression were done. RNA extraction was performed with Trizol (Invitrogen, Waltham, MA, USA). For the qPCR-based mRNA export analysis, all the reactions were performed with Takara SYBR Premix Ex Taq (Takara, Japan) according to the manufacturer’s instructions and quantified by a CFX96 Real-Time PCR System (Bio-Rad, Hercules, CA, USA). The relative fold changes were calculated using the 2(-Delta Delta C (T)) method. The primer pairs used for qPCR in this study are listed in Supplementary Table 1.

### Statistical analysis

Student’s *t*-test was performed to analyze the differences between two groups, whereas a one-way analysis of variance (ANOVA) test was used among more than three groups. Pearson analysis was performed for correlation analysis. Data were presented as the mean ± standard error of the mean, and *P* < 0.05 was considered to be statistically significant.

### Data availability statement

Publicly available datasets were analyzed in this study. These data can be found as follows: The RNA-seq transcriptome data was downloaded from The Cancer Genome Atlas (TCGA, https://tcga-data.nci.nih.gov/tcga) and GEO database (https://www.ncbi.nlm.nih.gov/geoprofiles/?term).

## RESULTS

### The expression analysis of PUSs in NSCLC

**Figure 1:**
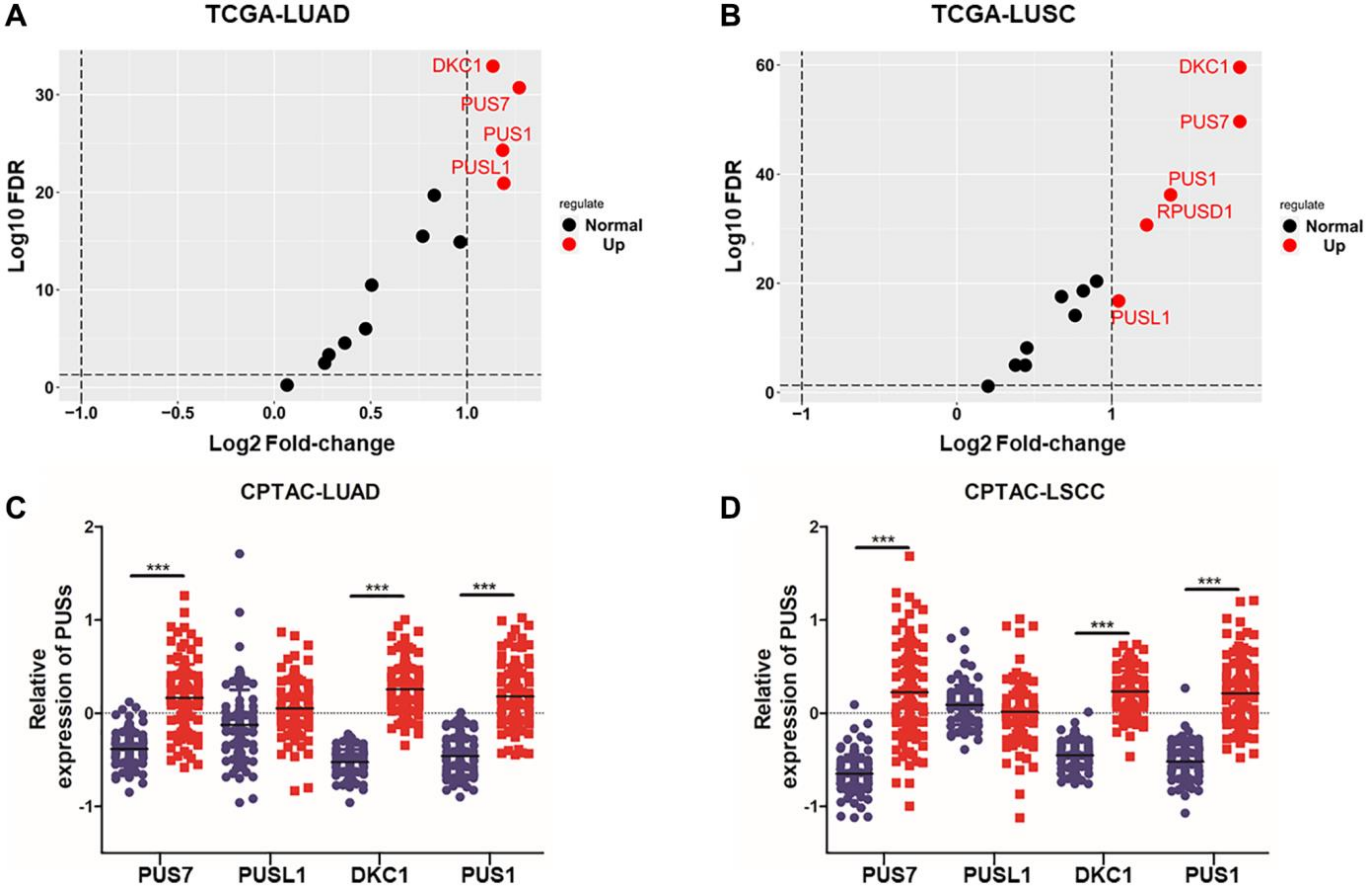
**The expression analysis of PUSs in NSCLC.** (**A**) The fold-change of 13 pseudouridine synthases in LUAD between cancer and normal tissues based on TCGA database; (**B**) The fold change of 13 pseudouridine synthases in LUSC between cancer and normal tissues based on TCGA database; (**C**) The protein expression of PUS1, PUS7, PUSL1 and DKC1 between normal tissue and primary tissue in LUAD was analyzed based on the CPTAC database; (**D**) The protein expression of PUS1, PUS7, PUSL1 and DKC1 between normal tissue and primary tissue in LSCC was analyzed based on the CPTAC database. ^*^P < 0.05, ^**^P < 0.01, ^***^P < 0.001.

To investigate the expression of pseudouridine synthases in NSCLC, we analyzed 13 pseudouridine synthases expression between cancer tissue and normal tissue based on the TCGA database (TCGA-LUAD, TCGA-LUSC). The fold change of 13 pseudouridine synthases in LUAD and LUSC is shown in Figure 1A, 1B. 5 of 13 pseudouridine synthases are up-regulated in LUSC and 4 in LUAD. PUS7, PUSL1, PUS1, and DKC1 are up-regulated in both LUAD and LUSC which fold-change >2. Besides, the protein expression between cancer tissue and normal tissue of PUS7, PUSL1, PUS1, and DKC1 in LUAD and LSCC were shown in Figure 1C, 1D. PUS7, PUS1, and DKC1 are significantly up-regulated in cancer tissue at both mRNA and protein levels. Therefore, multi-omics analysis revealed that PUS7, PUS1, and DKC1 are upregulated in NSCLC.

### The expression of PUS1 in NSCLC based on multiple databases

**Figure 2:**
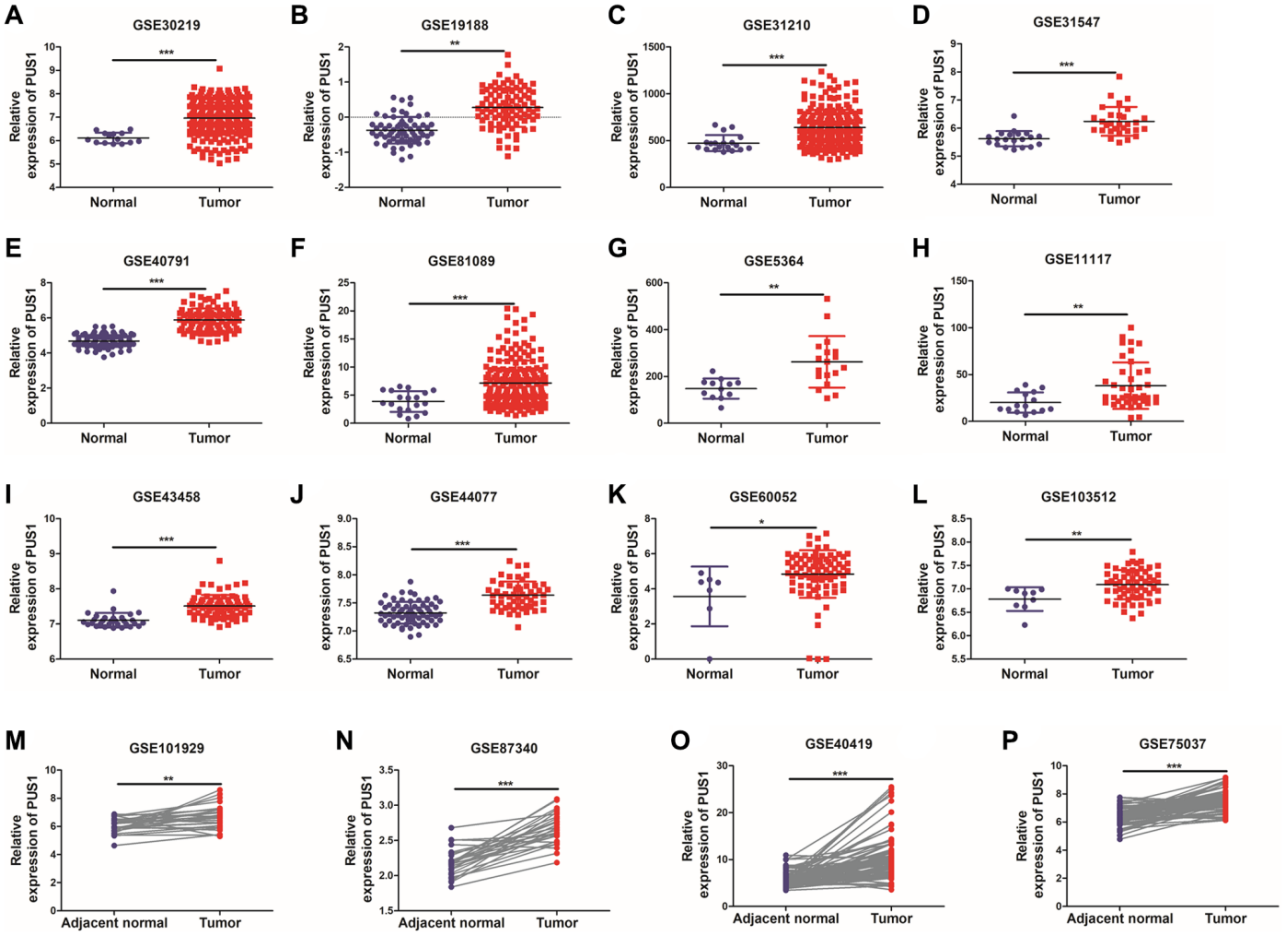
**The expression of PUS1 in NSCLC is based on multiple databases.** (**A**–**L**) The mRNA expression of PUS1 between normal tissue and primary tissue in NSCLC was analyzed based on GEO datasets (GSE30219, GSE19188, GSE31210, GSE31547, GSE40791, GSE81089, GSE5364, GSE11117, GSE43458, GSE44077, GSE60052, and GSE103512); (**M**–**P**) The expression of PUS1 in lung cancer and its matched adjacent normal tissues was obtained from GEO datasets. (GSE101929, GSE87340, GSE40419, and GSE75037). ^*^P < 0.05, ^**^P < 0.01, ^***^P < 0.001.

**Figure 3:**
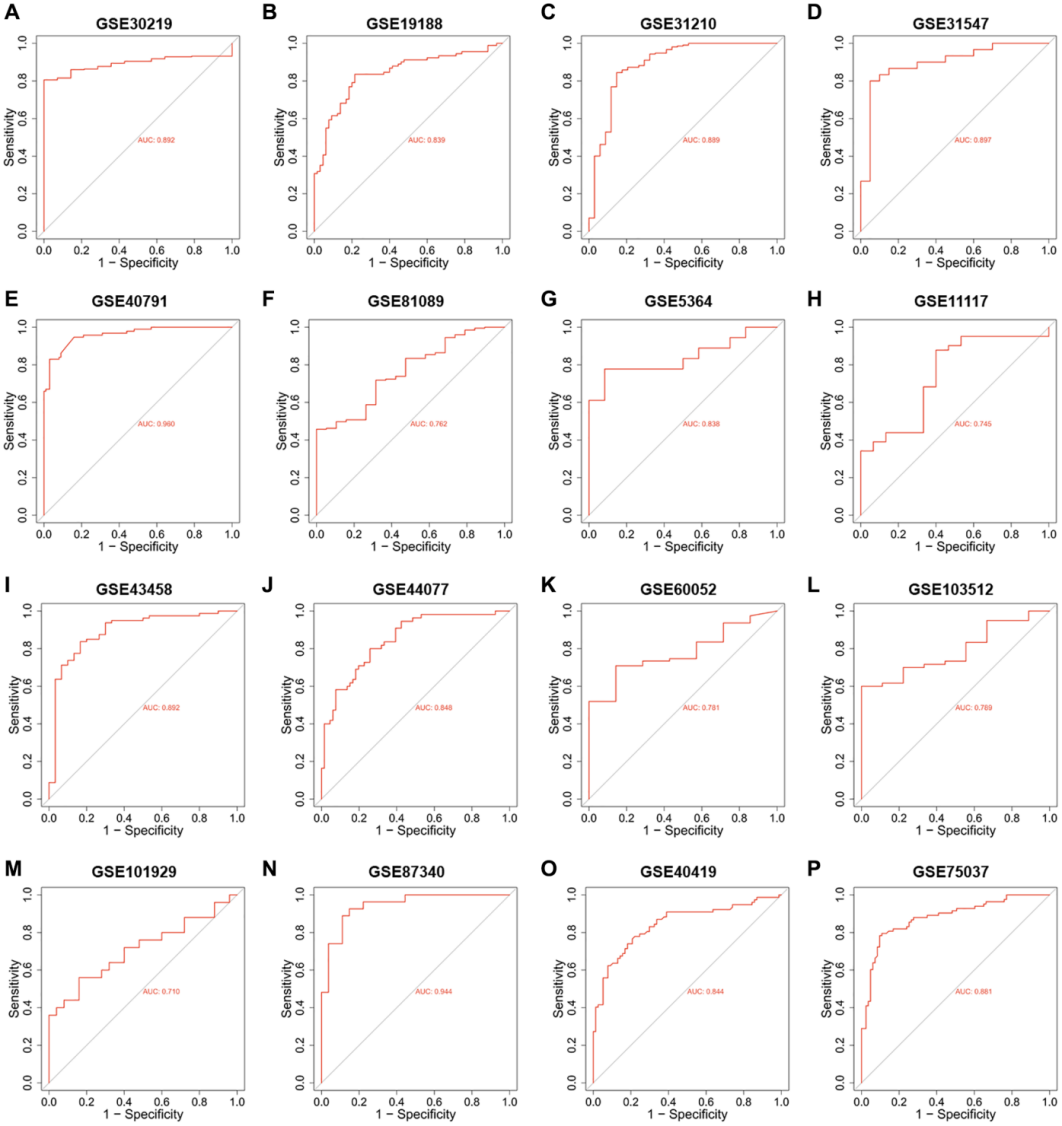
**The ROC analysis of PUS1 in NSCLC is based on multiple databases.** (**A**–**P**) The ROC analysis of PUS1 between normal tissue and primary tissue in NSCLC was analyzed based on GEO datasets. (GSE30219, GSE19188, GSE31210, GSE31547, GSE40791, GSE81089, GSE5364, GSE11117, GSE43458, GSE44077, GSE60052, GSE103512, GSE101929, GSE87340, GSE40419 and GSE75037).

Since the roles of PUS7 and DKC1 in NSCLC have been investigated, in this study, we focused on the role of PUS1 in the occurrence and development of NSCLC. A large number of studies on the GEO dataset in NSCLC can comprehensively analyze the role of PUS1 in NSCLC. We further analyzed the expression of PUS1 in NSCLC from the GEO database. The mRNA expression of PUS1 between normal tissue and primary tissue in NSCLC was analyzed based on GEO datasets (GSE30219, GSE19188, GSE31210, GSE31547, GSE40791, GSE81089, GSE5364, GSE11117, GSE43458, GSE44077, GSE60052, and GSE103512). These datasets indicated that PUS1 was significantly up-regulated in tumor tissue (Figure 2A–2L). The expression of PUS1 in lung cancer and its matched adjacent normal tissues of patients from GSE101929, GSE87340, GSE40419, and GSE75037 confirmed the results (Figure 2M–2P). The ROC analysis showed that the AUC of 16 GEO datasets ranges from 0.7–0.89 (Figure 3A–3P). In summary, the up-regulation of PUS1 expression in NSCLC has been confirmed by multiple GEO datasets.

### High expression of PUS1 promotes NSCLC development

**Figure 4:**
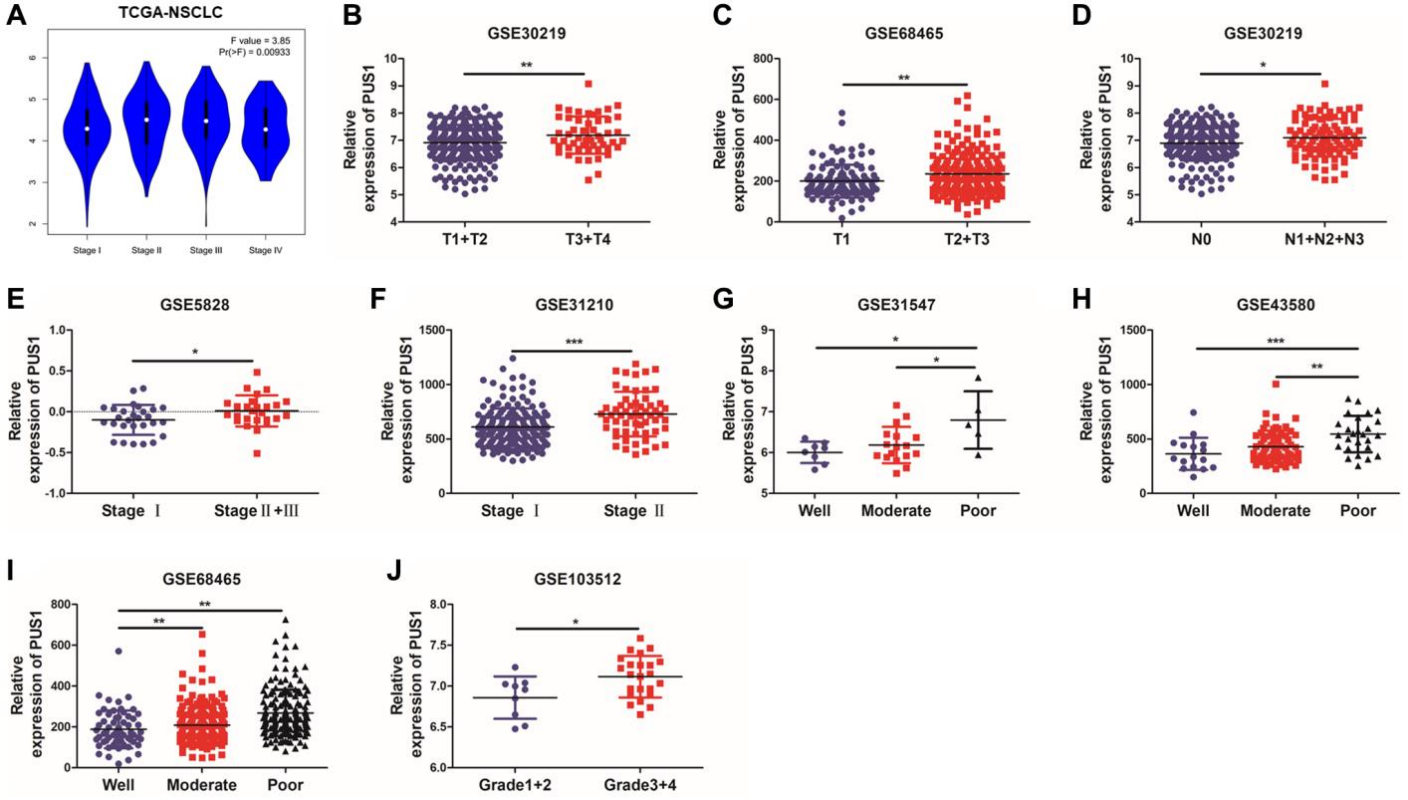
**High expression of PUS1 promotes NSCLC development.** (**A**) Based on the TCGA-LUAD and LUSC data, the expression of PUS1 was analyzed across the main pathological stages (stage I, II, III, and IV); (**B**, **C**) The correlation of PUS1 expression with different T stages in NSCLC based on the GSE30219 and GSE68465; (**D**) The correlation of PUS1 expression with different N stages in NSCLC is based on the GSE30219; (**E**, **F**) The correlation of PUS1 expression with different pathological stages in NSCLC is based on the GSE5852 and GSE31210; (**G**–**J**) The correlation of PUS1 expression with different differentiation in NSCLC is based on the GSE31547, GSE43580, GSE68465, and GSE103512. ^*^P < 0.05, ^**^P < 0.01, ^***^P < 0.001.

In order to determine the correlation between PUS1 and development in NSCLC, the relationship between PUS1 and tumor stage or differentiation was analyzed through multiple GEO datasets. Based on the TCGA-LUAD and LUSC data, the expression of PUS1 showed a significant difference among different main pathological stages (Figure 4A and Supplementary Figure 1A, 1B). Similarly, PUS1 expression was also significantly different among various T stage, N stage, and clinical stage based on GEO datasets. This result indicated that high expression of PUS1 correlates with larger tumor size (Figure 4B, 4C), higher lymph node metastasis (Figure 4D), and worse clinical stage (Figure 4E, 4F). In addition, tumor differentiation is closely related to the malignancy of the tumor. Poor differentiation predicts a worse prognosis. The expression of PUS1 was significantly up-regulated in patients with poor differentiation (Figure 4G–4J). In conclusion, high expression of PUS1 is correlated with the malignancy of NSCLC and may promote tumor development.

### High expression of PUS1 promotes NSCLC occurrence

**Figure 5:**
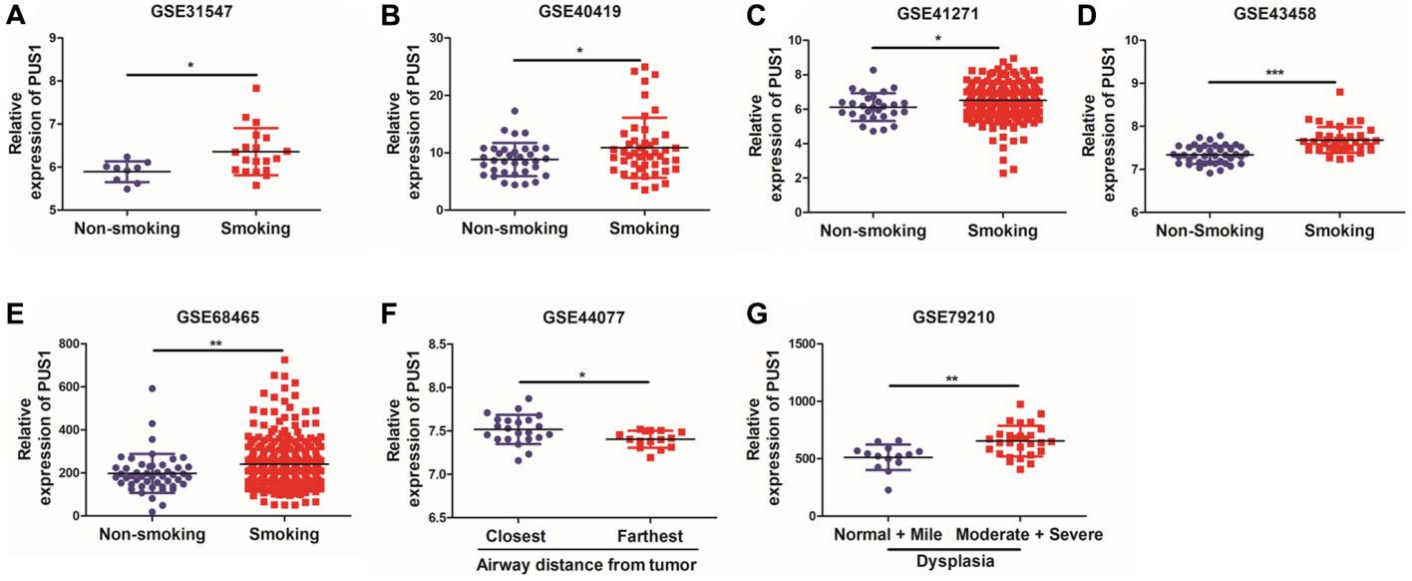
**High expression of PUS1 promotes NSCLC occurrence.** (**A**–**E**) The correlation of PUS1 expression and patients with smoking history in NSCLC based on the GSE31547, GSE40419, GSE41271, GSE43458, and GSE68465; (**F**) The correlation between PUS1 expression and the airway distance from the tumor in NSCLC was based on the GSE43580 dataset; (**G**) The correlation of PUS1 expression with different dysplasia in NSCLC based on the GSE79210.

Smoking history is recognized as an important prognosis factor in the occurrence of lung cancer. We further analyzed the correlation between the expression of PUS1 and patients’ smoking history. Interestingly, PUS1 was significantly up-regulated in patients with smoking history, which suggested that PUS1 may play a role in NSCLC occurrence. (Figure 5A–5E and Supplementary Figure 1C, 1D). In addition, we analyzed the correlation of PUS1 expression and the airway distance from tumor in NSCLC based on the GSE43580 (Figure 5F). The result has shown that PUS1 expression was higher in airway tissues near the tumor than in those far from the tumor. Besides, bronchial premalignant lesions (PMLs) were highly related to lung cancer. We also analyze the PUS1 expression in different dysplasia states. PUS1 was highly expressed in the moderate and severe dysplasia group compared to the normal and mile dysplasia group (Figure 5G). In summary, high expression of PUS1 may contribute to the occurrence of NSCLC.

### High expression of PUS1 affects the prognosis of NSCLC

**Figure 6:**
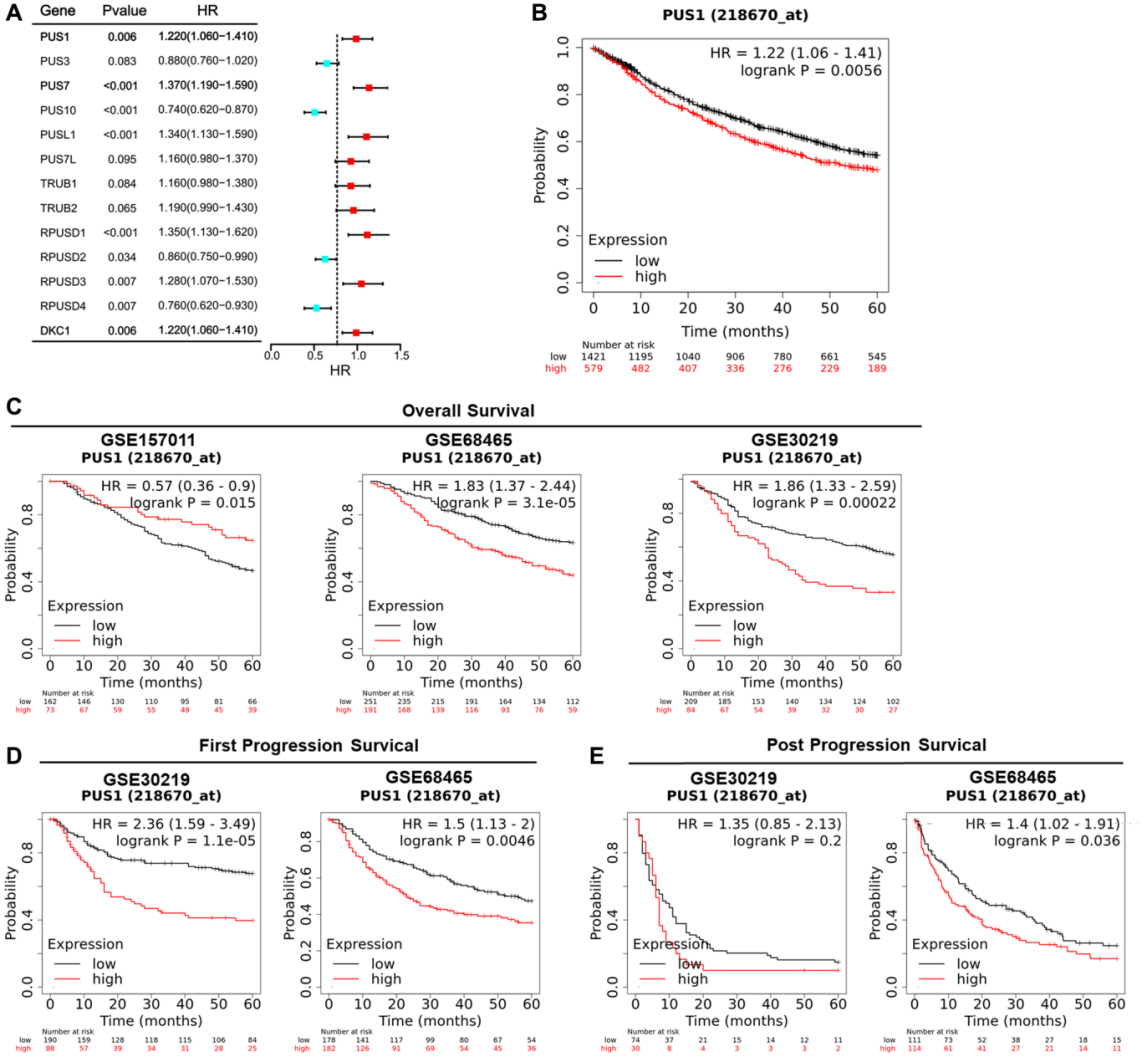
**High expression of PUS1 affects the prognosis of NSCLC.** (**A**) Univariate Cox regression analysis showing the hazard ratios (HRs) with 95% confidence intervals (CIs) and *P*-values for 13 PUSs; (**B**) The OS Kaplan-Meier survival curves comparing the high and low expression of PUS1 in NSCLC based on Kaplan-Meier plotter (17 datasets); (**C**) The OS Kaplan-Meier survival curves comparing the high and low expression of PUS1 in NSCLC based on GSE15701 (*N* = 235), GSE68465 (*N* = 442), and GSE30219 (*N* = 293), in Kaplan-Meier plotter; (**D**) The Kaplan-Meier survival curves for FPS comparing high and low expression of PUS1 in NSCLC were based on GSE68465 and GSE30219 in Kaplan-Meier plotter; (**E**) The PPS Kaplan-Meier survival curves comparing the high and low expression of PUS1 in NSCLC based on GSE68465 and GSE30219 in Kaplan-Meier plotter.

Survival analysis was conducted to examine the relationship between PUS1 expression and prognosis. Univariate Cox regression analysis had shown the hazard ratios (HRs) with 95% confidence intervals (CIs) and *P*-values for 13 PUSs based on the Kaplan-Meier plotter (Figure 6A and Supplementary Figure 2). The genes with HRs >1 and *P* < 0.05 included PUS1, PUS7, PUSL1, RPUSD1, RPUSD3 and DKC1. In addition, in the Kaplan-Meier plotter, which contains 17 NSCLC datasets, the OS Kaplan-Meier survival curves comparing the high and low expression of PUS1 indicated high expression of PUS1 predicts poor prognosis (Figure 6B). We then separately analyzed the GEO datasets with more than 200 patients to confirm that PUS1 is a prognostic factor in NSCLC. There are 3 datasets GSE15701 (*N* = 235), GSE68465 (*N* = 442), and GSE30219 (*N* = 293) involved, and 2 of them (GSE68465 and GSE30219) showed that high expression of PUS1 predicted poor prognosis (Figure 6C). Furthermore, compared to low expression levels, high expression levels of PUS1 were correlated with poorer FPS and PPS in NSCLC (Figure 6D, 6E). Overall, high expression of PUS1 affects the prognosis of NSCLC.

### The immune characteristics of PUS1 in NSCLC

**Figure 7:**
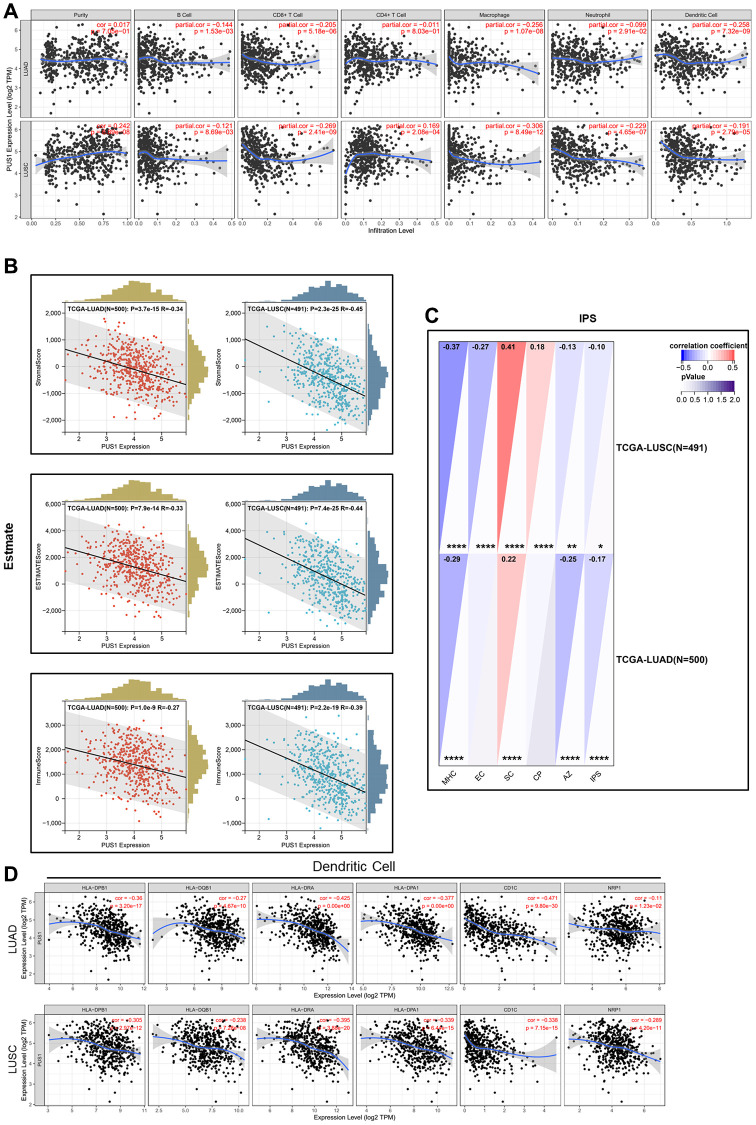
**The immune characteristics of PUS1 in NSCLC.** (**A**) Correlations of PUS1 expression with immune infiltration levels in LUAD and LUSC in TIMER; (**B**) Correlations of PUS1 expression with ESTIMATE score in LUAD and LUSC in ESTIMATE; (**C**) Correlations of PUS1 expression with IPS score in LUAD and LUSC in IPS; (**D**) The correlation analysis between PUS1 expression and gene markers of dendritic cell in LUAD and LUSC. ^*^P < 0.05, ^**^P < 0.01, ^***^P < 0.001.

Previous studies have shown that immune infiltration is an independent prognostic factor in tumors. Therefore, we investigated the correlation between PUS1 expression and immune infiltration levels in NSCLC. We performed the correlations of PUS1 expression with immune infiltration levels in LUAD and LUSC from TIMER. Results show that the expression of PUS1 has significant correlations with B cells, CD8+ T cells, macrophages, and Dendritic cells in LUAD. In LUSC, PUS1 has significant correlations with tumor purity, B cells, CD8+ T cells, CD4+ T cells, macrophages, neutrophils, and dendritic cells (Figure 7A). We also analyzed the stromal and immune cell levels by ESTIMATE. Results indicated that high expression of PUS1 was negatively correlated with stromal or immune scores in NSCLC (Figure 7B). IPS is a website to assess tumor immunogenicity, a higher IPS represents a more immunogenic tumor. In our study, we assessed the correlation between the expression of PUS1 and IPS. IPS analysis showed that PUS1 had a negative correlation with IPS in NSCLC (Figure 7C), which indicated that patients with high expression of PUS1 had less immunogenicity. In addition, we analyzed the correlation between PUS1 and immune cell markers based on TIMER (Table 1). We found that the expression of PUS1 was negatively correlated with dendritic cells marker genes. These results indicated that high expression of PUS1 predicted low levels of dendritic cells infiltration (Figure 7D). Low DC infiltration may cause tumor immune escape. In general, PUS1 was associated with immune cell infiltration.

**Table 1:** The correlation analysis between PUS1 and gene markers of immune cells in TIMER.

**Cell type**	**Gene marker**	**LUAD**				**LUSC**			
		**None**		**Purity**		**None**		**Purity**	
		**Cor**	***P***	**Cor**	***P***	**Cor**	***P***	**Cor**	***P***
CD8+Tcell	CD8A	0.014	7.49E-01	0.01	8.18E-01	−0.195	1.11E-05	−0.113	1.35E-02
	CD8B	0.029	5.10E-01	0.028	5.30E-01	−0.178	6.34E-05	−0.127	5.61E-03
Tcell (general)	CD3D	−0.093	3.51E-02	0.105	2.00E-02	−0.219	7.27E-07	−0.11	1.58E-02
	CD3E	−0.082	6.39E-02	−0.091	4.33E-02	−0.192	1.57E-05	−0.079	8.32E-02
	CD2	0.131	2.90E-03	0.145	1.29E-03	−0.225	3.41E-07	−0.123	7.10E-03
Bcell	CD19	0.003	9.41E-01	0.005	9.06E-01	−0.103	2.13E-02	0.036	4.30E-01
	CD79A	0.055	2.16E-01	−0.068	1.32E-01	−0.183	3.80E-05	−0.06	1.92E-01
Monocyte	CD86	−0.21	1.61E-06	−0.233	1.71E-07	−0.343	2.92E-15	−0.251	2.79E-08
	CD115 (CSF1R)	−0.208	1.84E-06	−0.251	1.39E-06	−0.278	2.46E-10	−0.173	1.51E-04
TAM	CCL2	−0.122	5.59E-03	−0.127	4.81E-03	−0.285	7.68E-11	−0.214	2.29E-06
	CD68	−0.112	1.09E-02	−0.122	6.47E-03	−0.295	1.58E-11	−0.214	2.34E-04
	IL10	−0.159	2.82E-04	−0.164	1.98E-04	−0.332	2.31E-14	−0.266	3.49E-09
M1Macrophage	INOS (NOS2)	0.099	2.51E-02	0.096	3.22E-02	0.07	1.20E-01	0.074	1.05E-01
	IRF5	0.019	6.70E-01	0.035	4.35E-01	0.11	1.37E-02	0.147	1.28E-03
	COX2 (PTGS2)	−0.022	6.14E-01	−0.038	4.02E-01	−0.137	2.14E-03	−0.101	2.71E-02
M2Macrophage	CD163	−0.105	1.67E-02	−0.111	1.37E-02	−0.282	1.27E-10	−0.194	1.97E-05
	VSIG4	−0.21	1.64E-06	−0.221	7.51E-07	−0.39	1.34E-19	−0.318	1.10E-12
	MS4A4A	−0.263	1.55E-09	−0.283	1.62E-10	−0.391	8.87E-20	−0.315	2.04E-12
Neutrophils	CD66b (CEACAM8)	−0.273	2.87E-10	−0.259	5.69E-09	−0.111	1.29E-02	−0.102	2.62E-02
	CD11b (ITGAM)	−0.174	7.20E-05	−0.173	1.10E-04	−0.14	1.63E-03	−0.029	5.31E-01
	CCR7	−0.158	3.24E-04	−0.158	4.23E-04	−0.122	6.32E-03	−0.003	9.49E-01
Naturalkillercell	KIR2DL1	0.112	1.08E-02	0.11	1.43E-02	0.073	1.01E-01	−0.041	3.70E-01
	KIR2DL3	0.107	1.56E-02	0.11	1.43E-02	−0.079	7.59E-02	−0.031	4.93E-01
	KIR2DL4	0.256	3.58E-09	0.257	6.92E-09	−0.1	2.47E-02	−0.037	4.20E-01
	KIR3DL1	0.093	3.40E-02	0.112	1.31E-02	−0.107	1.70E-02	−0.044	3.36E-01
	KIR3DL2	0.091	3.82E-02	0.099	2.75E-02	−0.038	4.02E-01	0.026	5.74E-01
	KIR3DL3	0.113	1.06E-02	0.122	6.69E-03	−0.022	6.25E-01	−0.004	9.26E-01
	KIR2DS4	0.032	4.73E-01	0.041	3.60E-01	−0.026	5.61E-01	0.006	8.97E-01
Dendriticcell	HLA-DPB1	**−0.36**	**3.20E-17**	**−0.386**	**5.44E-19**	**−0.305**	**2.97E-12**	**−0.205**	**6.17E-06**
	HLA-DQB1	**−0.27**	**4.67E-10**	**−0.285**	**1.09E-10**	**−0.238**	**7.28E-08**	**−0.149**	**1.10E-03**
	HLA-DRA	**−0.425**	**0.00E+00**	**−0.459**	**4.16E-27**	**−0.395**	**3.88E-20**	**−0.314**	**2.31E-12**
	HLA-DPA1	**−0.377**	**0.00E+00**	**−0.403**	**1.23E-20**	**−0.339**	**6.44E-15**	**−0.249**	**3.41E-08**
	BDCA-1 (CD1C)	**−0.471**	**9.80E-30**	**−0.471**	**1.23E-28**	**−0.338**	**7.15E-15**	**−0.246**	**5.15E-08**
	BDCA-4 (NRP1)	**−0.11**	**1.23E-02**	**−0.101**	**2.50E-02**	**−0.289**	**4.20E-11**	**−0.225**	**7.14E-07**
Th1	T-bet (TBX21)	0.042	3.40E-01	0.049	2.81E-01	−0.068	1.28E-01	0.044	3.40E-01
	STAT4	−0.11	1.28E-02	−0.117	9.30E-03	−0.212	1.74E-06	−0.114	1.26E-02
	STAT1	0.128	3.71E-03	0.134	2.91E-03	−0.069	1.25E-01	−0.011	8.08E-01
	IFN-γ (IFNG)	0.152	5.28E-04	0.16	3.49E-04	−0.057	2.01E-01	0.008	8.65E-01
	TNF-α (TNF)	−0.055	2.16E-01	−0.035	4.40E-01	−0.121	6.75E-03	−0.022	6.32E-01
Th2	GATA3	0.004	9.22E-01	0.017	7.12E-01	−0.059	1.87E-01	0.01	8.26E-01
	STAT6	0.006	8.92E-01	0.004	9.24E-01	0.204	1.27E-06	0.208	4.84E-06
	STAT5A	−0.088	4.50E-02	−0.077	8.93E-02	−0.123	6.81E-03	−0.024	9.59E-01
	IL13	0.016	7.14E-01	0.019	6.72E-01	−0.062	1.65E-01	−0.01	8.25E-01
Tfh	BCL6	−0.012	7.83E-01	0.003	9.46E-01	0.153	5.81E-04	0.129	4.90E-03
	IL21	0.081	6.66E-02	0.093	3.99E-02	−0.058	1.91E-01	0.013	7.78E-01
Th17	STAT3	0.079	7.29E-02	−0.081	7.23E-02	0.013	7.73E-01	0.044	3.34E-01
	IL17A	0.1	2.32E-02	0.11	1.48E-02	−0.018	6.81E-01	0.012	7.98E-01
Treg	FOXP3	0.023	6.10E-01	0.032	4.83E-01	−0.096	3.19E-02	0.029	5.26E-01
	CCR8	−0.081	6.56E-01	−0.07	1.19E-01	−0.2	6.42E-06	−0.097	3.46E-02
	STAT5B	0.027	5.35E-01	0.045	3.15E-01	0.143	1.33E-03	0.149	1.08E-03
	TGFβ (TGFB1)	−0.133	2.54E-03	−0.135	2.61E-03	−0.055	3.18E-01	0.006	8.89E+01
Tcell exhaustion	PD-1 (PDCD1)	0.17	1.09E-04	0.191	1.91E-05	−0.148	1.92E-01	0.005	9.68E-01
	CTLA4	0.067	1.27E-01	0.09	4.67E-02	−0.154	1.74E-01	−0.048	6.86E-01
	LAG3	0.166	1.61E-04	0.172	1.20E-04	0.09	4.30E-01	0.141	2.35E-01
	TIM-3 (HAVCR2)	−0.193	1.11E-05	−0.215	1.53E-06	−0.043	7.09E-01	0.084	4.78E-01
	GZMB	0.254	5.14E-09	0.269	1.31E-09	−0.107	3.48E-01	0.021	8.61E-01

### The potential downstream targets of PUS1

**Figure 8:**
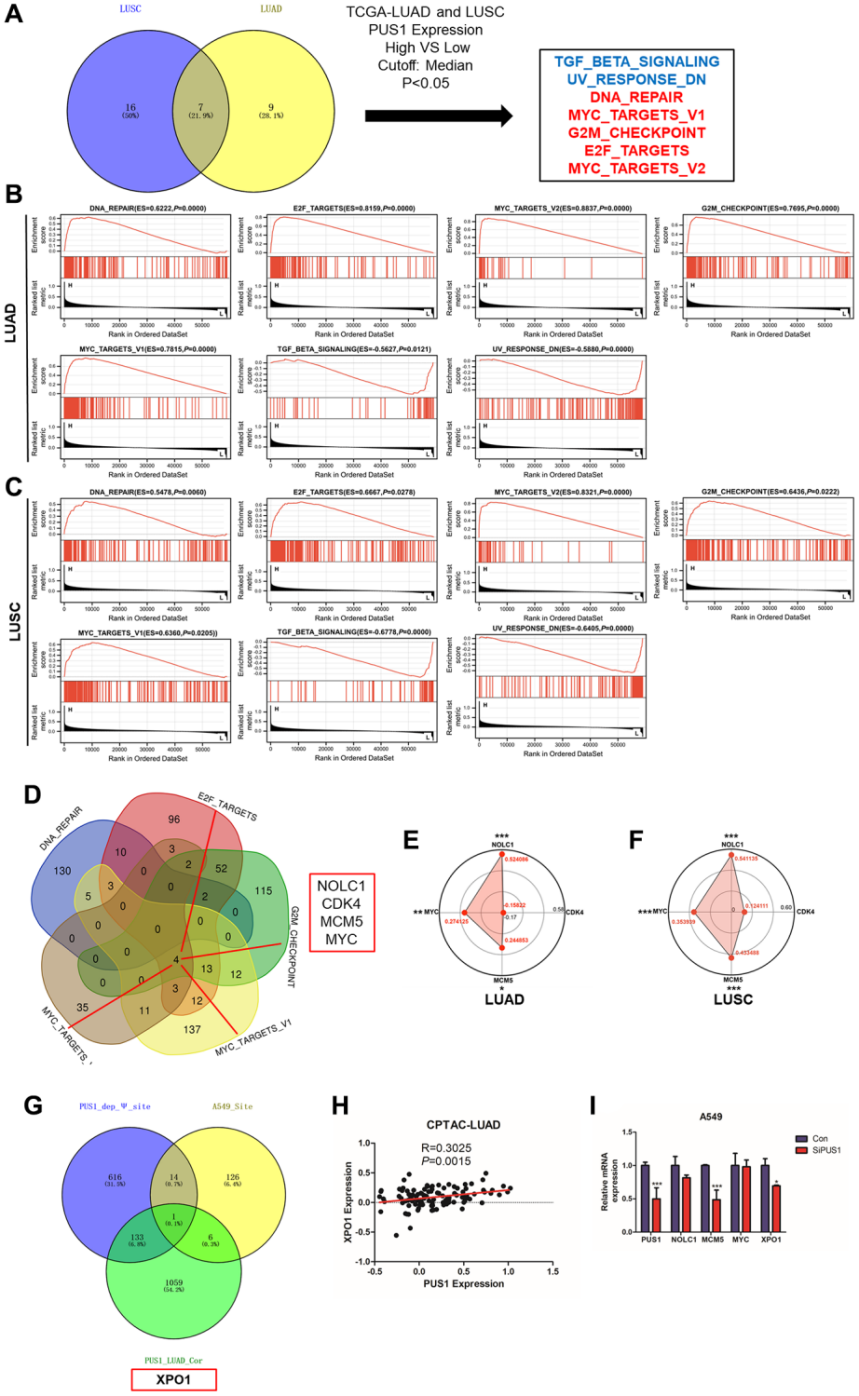
**The potential downstream targets of PUS1.** (**A**) The intersection analysis of the GSEA analysis in LUAD and LUSC between high– and low–PUS1 expression patients using hallmark gene sets; (**B**) GSEA analysis revealed that DNA_REPAIR, E2F_TARGETS, MYC_TARGETS_V2, G2M_CHECKPOINT, and MYC_TARGETS_V1 were significantly enriched in patients with high PUS1 expression, and TGF_BETA_SIGNALING, UV_RESPONSE_DN were significantly enriched in patients with low PUS1 expression in LUAD; (**C**) GSEA analysis revealed that DNA_REPAIR, E2F_TARGETS, MYC_TARGETS_V2, G2M_CHECKPOINT, and MYC_TARGETS_V1 were significantly enriched in patients with high PUS1 expression, and TGF_BETA_SIGNALING, UV_RESPONSE_DN were significantly enriched in patients with low PUS1 expression in LUSC; (**D**) The intersection analysis in DNA_REPAIR, E2F_TARGETS, MYC_TARGETS_V2, G2M_CHECKPOINT and MYC_TARGETS_V1 gene sets; (**E**, **F**) The correlation analysis between PUS1 expression and NOLC1, CDK4, MCM5, and MYC in LUAD and LUSS based on CPTAC dataset; (**G**) The intersection analysis in PUS1_dep_Ψsite, PUS1_LUAD_cor, and A549_ Ψ site; (**H**) The correlation analysis between PUS1 expression and XPO1 in LUAD based on CPTAC dataset. (**I**) The expression of NOLC1, MCM5, MYC, and XPO1 mRNA expression after knockdown of PUS1 in A549 cells. ^*^P < 0.05, ^**^P < 0.01, ^***^P < 0.001.

To further study the possible downstream and potential molecular mechanisms of PUS1 in NSCLC. GSEA analysis was performed between PUS1 high and low patients in LUAD and LUSC (Figure 8A, Supplementary Figure 3A, 3B). Results revealed that DNA_REPAIR, E2F_TARGETS, MYC_TARGETS_V2, G2M_CHECKPOINT, and MYC_TARGETS_V1 were significantly enriched in patients with high PUS1 expression, while TGF_BETA_SIGNALING, UV_RESPONSE_DN were significantly enriched in patients with low PUS1 expression in LUAD and LUSC (Figure 8B, 8C). There are 5 gene sets enriched in patients with high PUS1 expression. We performed an intersection analysis in DNA_REPAIR, E2F_TARGETS, MYC_TARGETS_V2, G2M_CHECKPOINT, and MYC_TARGETS_V1 to identify the potential targets of PUS1. Results showed that NOLC1, CDK4, MCM5, and MYC may be the potential targets of PUS1 (Figure 8D). We then confirmed the result through correlation analysis between PUS1 and 4 potential genes in the CPTAC database. We found PUS1 was significantly correlated with NOLC1, MCM5, and MYC in both LUAD and LUSC databases (Figure 8E, 8F). Furthermore, RT-qPCR verified the MCM5 decreased after the knockdown of PUS1 in the A549 cell (Figure 8I and Supplementary Figure 2).

The effect of PUSs on mRNA modification and the regulation of downstream gene expression is a current research hotspot. Another strategy was used to screen the downstream genes of PUS1. The intersection analysis in PUS1_dep_Ψ site, PUS1_LUAD_cor, and A549_ Ψ site found that XPO1 may be the potential target of PUS1 (Figure 8G). We analyzed the relationship between PUS1 and XPO1 in the CPTAC-LUAD database similarly. The result showed that PUS1 was significantly positively correlated with XOP1 (Figure 8H). Consistently, after the knockdown of PUS1, the expression of XPO1 significantly decreased (Figure 8I). In conclusion, PUS1 may be involved in DNA_REPAIR, E2F_TARGETS, MYC_TARGETS_V2, G2M_CHECKPOINT, and MYC_TARGETS_V1 pathways, potentially triggering NSCLC malignancy. MCM5 and XOP1 may be the potential targets of PUS1.

## DISCUSSION

Previous studies indicated that Ψ is one of the most abundant and widespread RNA modifications, and pseudouridine synthases have been found to be involved in NSCLC occurrence and development. In our study, multi-omics analysis revealed a significant increase in PUS1 expression in NSCLC, and high expression of PUS1 predicted a poor prognosis. Clinical subgroup analysis showed that PUS1 may be involved in the occurrence and development of NSCLC. Additionally, we analyzed the relationship between PUS1 and tumor immune infiltration. GSEA analysis also indicated that PUS1 may be involved in DNA_REPAIR, E2F_TARGETS, MYC_TARGETS_V2, G2M_CHECKPOINT, and MYC_TARGETS_V1 pathways, triggering NSCLC malignancy.

The roles of Ψ modification in RNA controlling the cancer progression is beginning to be unveiled. The initial report linking uridine modification to human disease was discovered through studies that specifically examined urinary metabolites in cancer patients [35]. Ψ was identified as a biomarker in NSCLC [15]. PUSs, as the ‘writers’ of Ψ, have been explored in many studies in recent years. DKC1 and PUS7 have been proven to be oncogenes in many cancers, such as colorectal cancer [16, 23], glioblastoma [18, 24], prostate cancer [19], breast cancer [21] and lung adenocarcinoma [22]. In our study, multi-omics analysis demonstrated a significant increase in PUS1 expression in tumor tissue compared to normal tissue, and high expression of PUS1 was indicative of a poor prognosis in NSCLC. NSCLC patients with a high expression of PUS1 had shorter OS, FPS, and PPS according to the Kaplan-Meier plotter database. PUS1 may be involved in the occurrence and development of NSCLC. High expression of PUS1 correlates with larger tumor size, more lymph node metastasis, a worse clinical stage, and poor differentiation. Consistently, previous studies in breast cancer and liver cancer have indicated PUS1 as an independent prognosis factor [25, 26]. The role of PUS1 in NSCLC was similar to PUS7 or DKC1. The increase of PUSs resulted in the upregulation of Ψ and correlated with malignancy.

Our analysis in TIMER and ESTIMATE suggested that PUS1 was associated with immune cell infiltration and in particular, it showed a significant negative correlation with DC cells in NSCLC. Moreover, decreased stromal and immune cell levels in high PUS1 expression patients demonstrated that tumor cells had a higher proportion in tumor tissues and immune cells infiltration was inhibited, potentially leading to a reduced effectiveness of immunotherapy drugs [36]. DC cells are important antigen-presenting cells in the tumor microenvironment that can help the immune system suppress tumor growth [37–39]. In addition, the IPS is an assessment of tumor immunogenicity based on four major categories of genes that determine immunogenicity [34]. The patients with high expression of PUS1 had lower IPS, which indicated low levels of immunogenicity and conferred tumor evasion from immune responses. These results showed that PUS1 expression may reduce DC cells infiltration in tumor tissue which can promote immune escape by tumor cells. Taken together, high expression of PUS1 suppresses the enrichment of antigen-presenting cells, leading to a low response ratio to immunotherapy treatment and increased infiltration of tumor cells.

Ψ regulates the expression of downstream genes to affect the occurrence and development of tumors is a prominent subject of research. Studies are currently focused on developing novel and reliable methods for detecting Ψ to identify potential downstream targets. PUS1, one of the “writers” of Ψ, was involved in the occurrence and development of NSCLC in our study. Therefore, we employed two strategies to identify potential targets of PUS1. GSEA analysis revealed that DNA_REPAIR, E2F_TARGETS, MYC_TARGETS_V2, G2M_CHECKPOINT, and MYC_TARGETS_V1 were significantly enriched in patients with high PUS1 expression. We performed an intersection analysis in DNA_REPAIR, E2F_TARGETS, MYC_TARGETS_V2, G2M_CHECKPOINT, and MYC_TARGETS_V1, and along with RT-QPCR, to determine whether MCM5 could be a potential target of PUS1. Previous studies have also shown that PUS7 and DCK1 regulation of MYC expression promotes tumor malignancy.

In addition, another intersection analysis between PUS1_dep_Ψsite, PUS1_LUAD_cor, and A549_ Ψ suggested that XPO1 may be a potential downstream target affected by Ψ. Studies suggest that dysregulation of XPO1 promotes progression in several tumors. XPO1 is also one of the targets for tumor therapy. Our study suggested the knockdown of PUS1 decreased the expression of XPO1. These findings suggest that PUS1, and potentially Ψ, may play a role in triggering the malignancy of NSCLC through involvement with XPO1.

However, there were limitations to our study. Firstly, systematic bias may have been introduced due to the large proportion of microarray and sequencing data used in our study. Secondly, the exact role and mechanism of PUS1 in immunotherapy also need further study. Thirdly, this study only conducted a bioinformatics analysis of PUS1 expression and patient prognosis based on multi-omics NSCNC databases. The molecular mechanism of PUS1 regulating MCM5 or XPO1 expression still requires further experiments. Our results may become more reliable if *in vitro* and *in vivo* experiments are performed in our research.

In conclusion, our study based on multi-omics analysis revealed a significant increase of PUS1 in NSCLC and high expression of PUS1 predicted a poor prognosis. Clinical subgroup analysis showed that PUS1 may play a role in the occurrence and development of NSCLC. Besides, we analyzed the relationship between PUS1 and tumor immune infiltration. GESA analysis also indicated PUS1 may be involved in DNA_REPAIR, E2F_TARGETS, MYC_TARGETS_V2, G2M_CHECKPOINT, and MYC_TARGETS_V1 pathways, potentially triggering NSCLC malignancy. Overall, PUS1 may be a potential therapy target and novel biomarker in NSCLC.

## Supplementary Materials

Supplementary Figures

Supplementary Table 1
