# Supplementary material for: Multi-omics analysis reveals PUS1 triggered malignancy and correlated with immune infiltrates in NSCLC
Source: Aging (Albany NY). 2023 Nov 2;15(21):12136–54. doi: 10.18632/aging.205169 (PMC10683629; doi:10.18632/aging.205169)
Supplement: Supplementary Figures [file aging-15-205169-s001.pdf]

SUPPLEMENTARY FIGURES

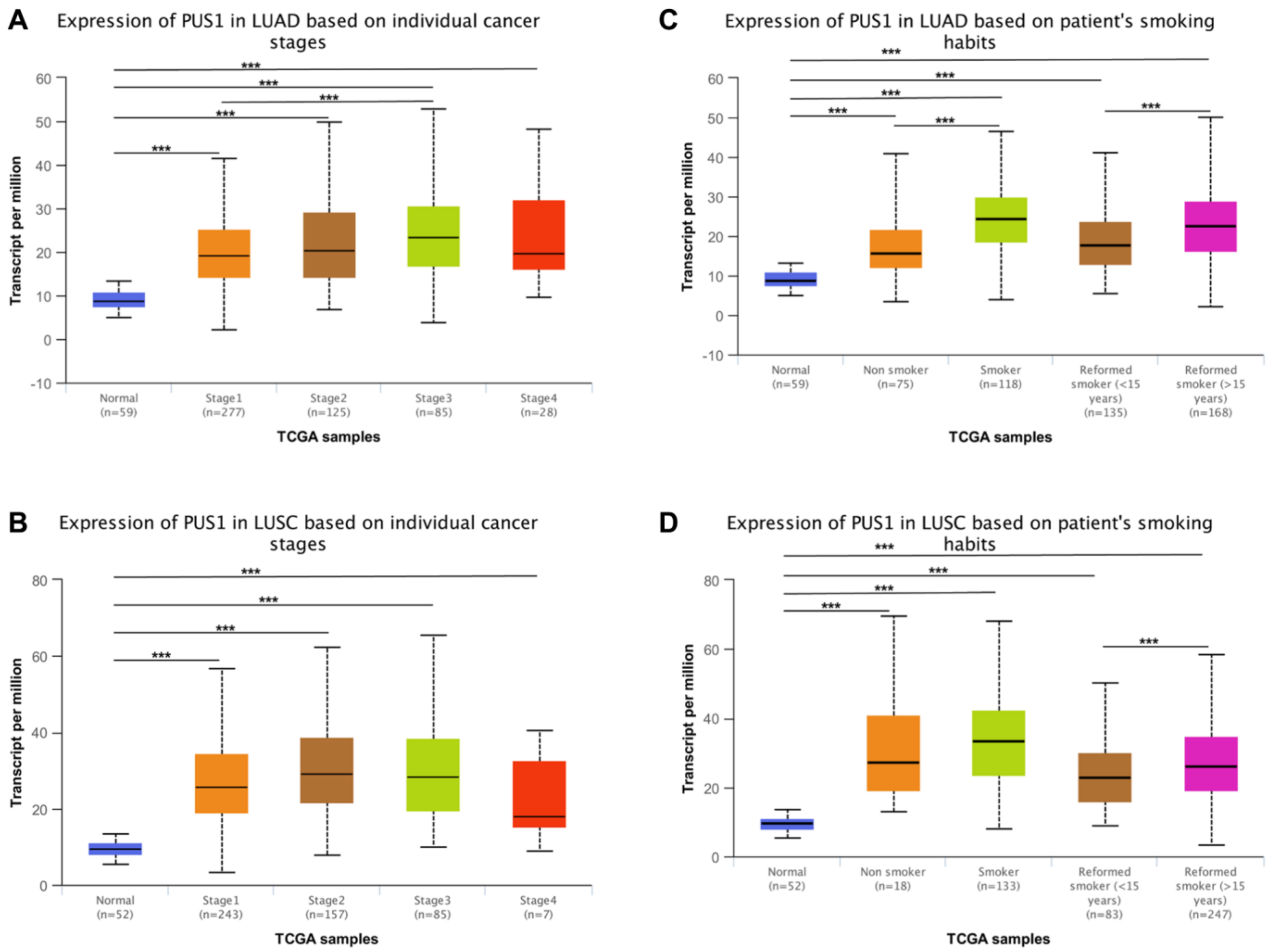

Supplementary Figure 1. (**A**, **B**) The relationship between PUS1 and stage according to TCGA-LUAD (**A**)/LUSC (**B**); (**C**, **D**) The relationship between PUS1 and smoking habits according to TCGA-LUAD (**C**)/LUSC (**D**).

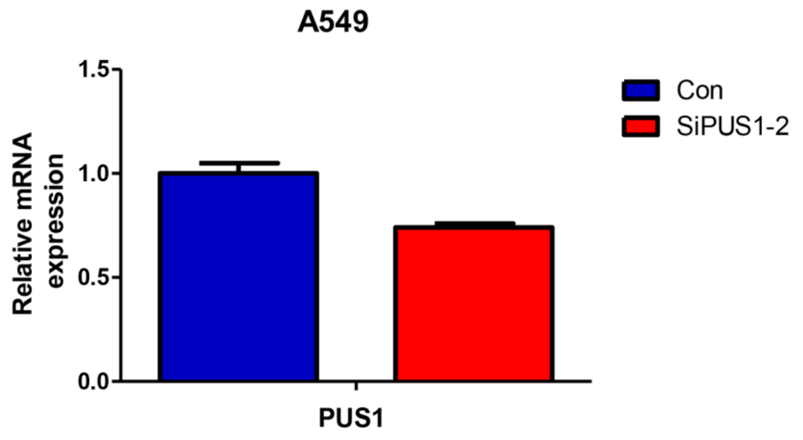

Supplementary Figure 2. The PUS1 expression after knock-down with PUS1-Si2 by RT-qPCR.

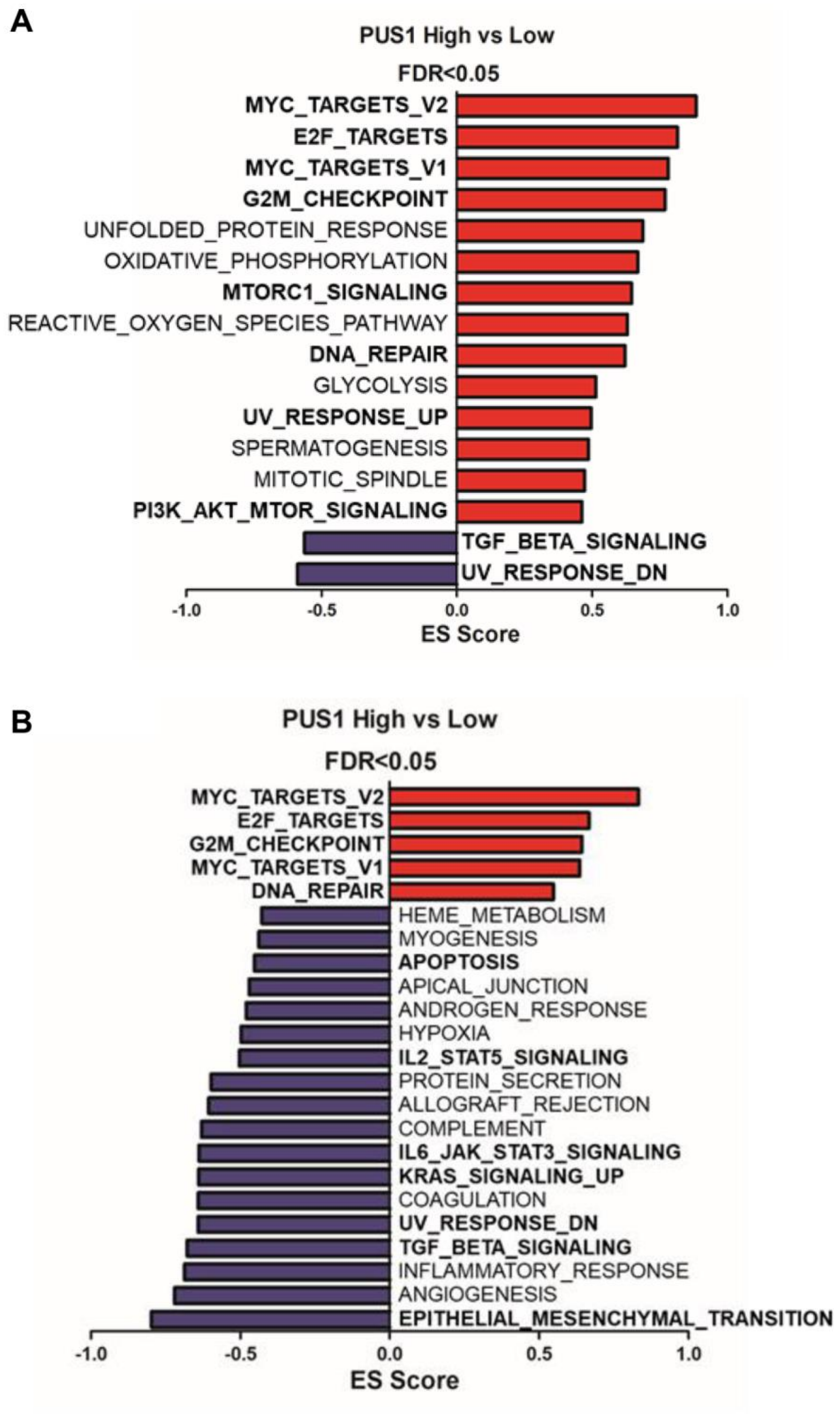

Supplementary Figure 3. (**A**, **B**) The GSEA analysis between PUS1 high and low patients in LUAD and LUSC (FDR < 0.05, Cutoff: median).
