# Supplementary material for: Multi-omics analysis reveals PUS1 triggered malignancy and correlated with immune infiltrates in NSCLC
Source: Aging (Albany NY). 2023 Nov 2;15(21):12136–54. doi: 10.18632/aging.205169 (PMC10683629; doi:10.18632/aging.205169)
Supplement: Supplementary Table 1 [file aging-15-205169-s002.pdf]

SUPPLEMENTARY TABLE

Supplementary Table 1. The primer pairs used for qPCR.

PUS1	F	CACGGGCGGGTTTAACTCCAAG
	R	GCTCAGGCGGTAGGTCTCATCC
NOLC1	F	GTAGCAGTGATGACTCAGAGGAG
	R	CTGGAGGAATCCTCACTGCTAG
MCM5	F	GACTTACTCGCCGAGGAGACAT
	R	TGCTGCCTTTCCCAGACGTGTA
MYC	F	CCTGGTGCTCCATGAGGAGAC
	R	CAGACTCTGACCTTTTGCCAGG
XPO1	F	CTACATCTGCCTCTCCGTTGCT
	R	CCAATACTTCCTCTGGTTTAGCC
